# Factors Influencing Pedestrian Smartphone Use and Effect of Combined Visual and Auditory Intervention on “Smombies”: A Chinese Observational Study

**DOI:** 10.3389/ijph.2022.1604601

**Published:** 2022-06-22

**Authors:** Qing-hong Hao, Yang Wang, Ming-Ze Zhou, Ting Yi, Jia-Rui Cui, Ping Gao, Mi-Mi Qiu, Wei Peng, Jun Wang, Yang Tu, Ya-Lin Chen, Hui Li, Tian-Min Zhu

**Affiliations:** ^1^ School of Rehabilitation and Health Preservation, Chengdu University of Traditional Chinese Medicine, Chengdu, China; ^2^ School of Traditional Chinese Medicine, Chongqing Medical University, Chongqing, China; ^3^ School of Acupuncture and Tuina, Chengdu University of Traditional Chinese Medicine, Chengdu, China; ^4^ School of Preclinical Medicine, Chengdu University, Chengdu, China

**Keywords:** smartphone use, smombie, visual and auditory intervention, pedestrian, observational study

## Abstract

**Objective:** This was a large-scale multicenter study with two objectives. One was to study the factors influencing pedestrian smartphone use while crossing roads, and the other was to study the effect of combined visual and auditory intervention on smartphone zombies (smombies) at crossroads.

**Methods:** This study was conducted in four different Chinese cities. By observing pedestrians crossing intersections, the weather, time, and characteristics of the pedestrians were recorded by four researchers. Then, its influencing factors and the effects of the intervention were studied in two consecutive periods.

**Results:** A total of 25,860 pedestrians (13,086 without intervention and 12,774 with visual and auditory intervention) were observed in this study. Logistic regressions showed that gender, age of the pedestrians, weather, and time were the factors influencing smombies crossing roads. The number of smartphone users decreased from 4,289 to 3,579 (28.1%) (*χ* ^*2*^ = 69.120, *p* < 0.001) when the intervention was conducted.

**Conclusion:** Based on large-sample, multicenter research, this study revealed the factors influencing pedestrian smartphone use while crossing roads, contributing to our understanding of the current situation of smombies in China. Furthermore, the effect of visual and auditory intervention was demonstrated, providing a new paradigm for global prevention of smombie behavior.

## Introduction

With the rapid advancement of the mobile internet, the functions of smartphones are becoming increasingly diversified, making smartphones indispensable in people’s work and lives. However, the massive use of smartphones is a double-edged sword. The term “smombie”, derived from “smartphone” and “zombie”, refers to pedestrians who use smartphones while walking [1]. Gazing at smartphones, smombies cannot perceive their surroundings, which is especially dangerous while crossing roads. However, the phenomenon of pedestrian smartphone use while crossing roads is common in many regions worldwide [2–4].

In Beijing, the percentage of pedestrian smartphone use while crossing varied from 11.7% to 21.8% [5], while in Seattle, the percentage was as high as one-third [6]. Due to the high smombie rates, it has become an emerging safety concern [7]. Pedestrians who use smartphones are always distracted, which increases the risk of negative consequences in traffic [1, 8–10]. An experimental study showed that 75% of pedestrians using smartphones while crossing did not response to visual stimuli [11]. Thus, it is not surprising that traffic accidents caused by distracting smartphone use have increased steeply in recent years [12–14].

The phenomenon of smombieism has attracted the concerns of researchers around the world. In addition to the prevalence of smombieism [5, 6], many studies have focused on the influencing factors of pedestrian smartphone use while walking [2, 6, 15–17]. Schaposnik et al. [2] explored the influence of gender on smartphone use while walking and found that women used smartphones while walking more frequently than men. In addition, age was also supposed to be a significant factor influencing smartphone use while crossing roads [6, 15, 17]. Fernández et al. [1] found that teenagers were the main group using smartphones while walking. However, all these studies were small-sample and single-center studies. Large-sample and multicenter studies could provide unbiased and empirical evidence of factors influencing smombies, but are still lacking. The study of Barin et al. [18] confirmed that visual intervention significantly affected pedestrian smartphone use in the short term. Violano et al. [19] found that pedestrians’ smartphone use behavior differed at intersections with visual reminders compared to intersections without reminders. Based on these findings, it is critical to explore potential preventive strategies against smombieism to better protect public health [18, 20].

Therefore, the current study aims to explore the factors influencing pedestrian smartphone use and the effect of a potential intervention on smombies while crossing roads. Four cities (Changchun, Lanzhou, Zhuzhou, and Luzhou) in different parts of China were selected as research sites to observe pedestrian smartphone use while crossing roads. In addition to the gender and age of the pedestrians, weather and time were also studied as potential factors in the first 11 days without intervention. In the next 11 days, combined visual and auditory intervention was taken to prevent pedestrians from using their smartphone while crossing the road. We hypothesized that ① variables influencing whether pedestrians are smombies while crossing the road include age, gender, weather, and time, and that ② visual and auditory intervention will prevent pedestrians from using smartphones while crossing the road.

## Methods

Here, we conducted a large-sample and multicenter observational study to explore the factors influencing pedestrian smartphone use and the effect of the potential intervention on smombies while crossing roads. Observing the pedestrians crossing the intersections, the factors influencing smartphone use while crossing the road were studied in the first 11 days without intervention. Then, visual and auditory intervention was performed over the next 11 days. The differences in pedestrian smartphone use between these two periods were compared to study the effect of visual and auditory intervention on smombies at crossroads.

### Research Sites

In the present study, the following four cities located in different parts of China were selected as research sites: Changchun (in northeast China), Lanzhou (in northwest China), Zhuzhou (in central China), and Luzhou (in southwest China). A crossroad near a residential area was randomly selected in each city.

### Research Time

The study was conducted from 19 August 2020 to 9 September 2020, in the four selected cities. The observation time was from Monday to Sunday, 07:30–09:00 in the morning and 17:00–18:30 in the afternoon. Days with extremely bad weather such as heavy rain, hail, and gale were excluded.

### Pedestrian Eligibility

#### Inclusion Criteria

The following pedestrians were included:

1) Pedestrians openly using smartphones while crossing a road who were defined as smombies;
2) Pedestrians passing through the fixed zebra crossings (crossing both the first and last standard zebra crossings lines);
3) Including but not limited to those jogging or walking (including those leading pets or pushing strollers);
4) Only the accompanying person was included for wheelchair users with an accompanying person.

A fixed zebra sign is shown in Supplementary Figure S1.

#### Exclusion Criteria

The following pedestrians were excluded:

1) Those who wore a watch (it is difficult for observers to judge whether the watch is a smartwatch);
2) Bike riders or skateboarders;
3) People who had severe visual disabilities (such as blind people);
4) People at work (such as delivery persons, mail delivery persons, and sanitation workers);
5) Other unsafe behaviors while crossing the road, such as reading or looking for things with their head down.

### Classification of Smartphone Use

By drawing on and slightly modifying the existing research [2, 21], this study divided the behaviors of pedestrian smartphone use into the following six categories, from low to high use (see Supplementary Figure S2):

1) Not observed: The pedestrians did not openly carry or use smartphones;
2) Talking: The pedestrians were making calls in a traditional way by holding the smartphones in their hands close to their ears and mouths;
3) Earphones: The pedestrians were wearing earphones;
4) Holding: The pedestrians had their smartphones in hand while crossing the road, but were not looking at the smartphones;
5) Slight smombie: The pedestrians were looking at smartphone screens, but were not operating the smartphone screens directly;
6) Severe smombie: The pedestrians were interacting with smartphones while crossing, such as operating the smartphones, typing on the screens, or using other functions of the smartphones.

The pedestrians with two established behaviors (or mixed categories) simultaneously were placed in the higher use category. For example, pedestrians wearing earphones and holding smartphones simultaneously were classified as “holding”.

### Classification of Variable Characteristics

The researchers registered the gender and approximate age of the pedestrians based on perceived appearance. Gender was divided into the following two categories: male and female. The weather was divided into the following three categories: sunny, cloudy, and rainy. The time of day was divided into morning and afternoon. The time of the week was divided into weekdays and weekends. For the age of the pedestrians, five classes were used as follows:

1) Under 10 years old: children aged 4–10;
2) Aged 11–25: teenagers;
3) Aged 26–44: young people;
4) Aged 45–59: middle-aged people;
5) Over 60 years old: older people.

### Intervention Measures

To study the effect of the intervention on preventing smartphone use while crossing the road, we intervened with pedestrians in the last 11 days. It should be pointed out here that the pedestrians who were study subjects on the 11 days of intervention did not necessarily coincide with those observed during the first 11 days. Considering smartphone distraction, this study proposed a combined visual and auditory measure to prevent pedestrians from using smartphones while crossing the road.

1) Safety warning signs

The “no smartphone” sign was placed at the intersections to remind pedestrians not to use smartphones while crossing the road. The safety warning sign is shown in Supplementary Figure S3.

2) Sound alerts

A broadcasting device was placed near the safety sign, repeating “do not use smartphones while crossing the road” (100 dB, loop playback) in Chinese, to remind pedestrians in an auditory way.

### Pedestrian Observation Procedure

In this study, we had four researchers, one in each city, record the circumstances surrounding pedestrians using smartphones while crossing a road. We recorded smombie gender and age, the time and weather, and the types of smartphone use. Only the pedestrians who passed the preset zebra crossings and crossed the road in a specific direction were recorded. Pedestrians walking alone or in a group of four people or less were observed directly, while pedestrians in a group of more than four were videoed [6, 22] because of potential counting errors [1].

Researchers quickly registered the digital codes while observing. Male pedestrians were marked as 1, and female pedestrians were marked as 2. The different weather conditions were classified as 1, 2, and 3; the age groups were divided into 1, 2, 3, 4, and 5; and the different types of smartphone users were recorded as 1, 2, 3, 4, 5, and 6. For example, for a 23-year-old man wearing earphones while crossing a road on a sunny day, the code was 1231. In addition, the morning was marked as 1, while the afternoon was marked as 2; the weekday was marked as 1, while the weekend was marked as 2.

In pre-observation, Kendall’s W test was used to check the consistency of results from the four different researchers. The results verified the consistency among researchers (W > 0.7, *p* < 0.001). This process was performed by a supervisor other than the four researchers.

All procedures of this study were approved by the ethics committee of the affiliated hospital of Chengdu University of Traditional Chinese Medicine (ethical approval number 2016 KL-005), in accordance with the Declaration of Helsinki.

### Study Size

Previous studies have shown that the proportion of pedestrian smartphone use is approximately 30% [5, 6]. If δ≤ 6%, *α* = 0.05, the required sample size calculated by PASS software (version 15.0) is 2543. The formula is as follows: $n=\frac{U_{\alpha }^{2}p\left(1-p\right)}{\delta^{2}}$ .

### Statistical Methods

All statistical analyses were performed with SPSS (version 25.0) software. All variables were considered categorical (such as age, divided into five age groups). Bivariate and multivariate logistic regression analyses were used to analyze the impacts of the influencing factors on pedestrian smartphone use (two categories/multiple categories) and the effect of the combined visual and auditory intervention. In addition, chi-square tests were used to evaluate the significant differences with and without intervention.

## Results

### Descriptive Statistics

A total of 25,860 pedestrians (13,086 without intervention and 12,774 with intervention) were observed in this study. The detailed descriptive statistical results of this study are shown in Table 1.

**TABLE 1:** Descriptive statistics of this study (N = 25,680). (Original research, smombie phenomenon, China, 2020).

	Without intervention (*n* = 13,086)	With intervention (*n* = 12,774)
Gender		
Male	5,965 (45.6%)	5,782 (45.3%)
Female	7,121 (54.4%)	6,992 (54.7%)
Age		
<10 years	516 (3.9%)	1,491 (11.7%)
11–25 years	2,317 (17.7%)	1,369 (10.7%)
26–44 years	5,716 (43.7%)	5,720 (44.8%)
45–59 years	2,908 (22.2%)	2,745 (21.5%)
>60 years	1,629 (12.4%)	1,449 (11.3%)
Region		
Changchun	3,909 (29.9%)	3,456 (27.1%)
Lanzhou	3,187 (24.4%)	3,788 (29.7%)
Zhuzhou	3,165 (24.2%)	2,679 (21.0%)
Luzhou	2,825 (21.6%)	2,851 (22.3%)
Weather		
Sunny	10,491 (80.2%)	10,327 (80.8%)
Cloudy	1,703 (13.0%)	1,292 (10.1%)
Rainy	892 (6.8%)	1,155 (9.0%)
Time		
Morning	5,853 (44.7%)	6,178 (48.4%)
Afternoon	7,233 (55.3%)	6,596 (51.6%)
Weekday	9,672 (73.9%)	9,255 (72.5%)
Weekend	3,414 (26.1%)	3,519 (27.5%)

### Smartphone Use Without Intervention

By analyzing the usage rate of smartphones without intervention, it was found that women were more likely to use smartphones while crossing roads (*χ* ^*2*^ = 120.905, *p* < 0.001); smartphone use while crossing roads was most prevalent among 11 to 25-year-old people, followed by young people aged 24–44 (See Supplementary Table S1); smartphone use while crossing roads was less prevalent on rainy days in terms of weather (See Supplementary Table S2); smartphone use was more prevalent in the afternoon than in the morning (*χ* ^*2*^ = 41.190, *p* < 0.001), and was more prevalent on weekdays than on weekends (*χ* ^*2*^ = 16.218, *p* < 0.001). See Table 2.

**TABLE 2:** Bivariate logistic regression analysis of pedestrian smartphone use. (Original research, smombie phenomenon, China, 2020).

Categorical Variable	Usage Rates (%)	Wald χ^2^	*P*	Odds Ratio (OR)	95% CI of OR	
					Lower	Upper
Male	23.2	47.943	**<0.001**	0.757	0.699	0.819
Female	32	Ref.	Ref.	Ref.	Ref.	Ref.
<10 years	0.5	6.573	**0.010**	0.495	0.289	0.847
11–25 years	42.4	635.325	**<0.001**	16.663	13.389	20.738
26–44 years	40.6	472.932	**<0.001**	10.248	8.309	12.639
45–59 years	20	184.226	**<0.001**	4.599	3.689	5.732
>60 years	8.4	Ref.	Ref.	Ref.	Ref.	Ref.
Sunny	27.8	49.519	**<0.001**	1.786	1.519	2.099
Cloudy	33.0	36.541	**<0.001**	1.781	1.477	2.147
Rainy	24.8	Ref.	Ref.	Ref.	Ref.	Ref*.*
Morning	26.4	7.921	**0.005**	0.890	0.820	0.965
Afternoon	29.5	Ref.	Ref.	Ref.	Ref.	Ref.
Weekday	29.1	13.874	**<0.001**	1.187	1.085	1.299
Weekend	25.1	Ref.	Ref.	Ref.	Ref.	Ref.

Ref.: reference variable. The meaning of the bold values is that they are statistically significant.

Further analysis of different types of pedestrian smartphone use revealed the distribution of different conditions. The “holding” rates were higher in women than in men (*χ* ^*2*^ = 157.348, *p* < 0.001). Teenagers had the highest “earphone”, “holding”, “slight smombie”, and “severe smombie” rates among all age groups, and the “talking” rates in both the teenage and young people groups were much higher than those in the other groups (See Supplementary Table S3). On rainy days, the rates of “holding” and “severe smombie” were the lowest (See Supplementary Table S4). “Talking” (*χ* ^*2*^ *=* 10.544, *p* < 0.01) and “severe smombie” (*χ* ^*2*^ = 19.505, *p* < 0.001) were more prevalent in the afternoon than in the morning. See Table 3.

**TABLE 3:** Multivariate logistic regression analysis of pedestrian smartphone use. (Original research, smombie phenomenon, China, 2020).

	Usage rates	Wald χ^2^	*P*	OR	95% CI of OR	
					Lower	Upper
Talking						
Male	2.2%	0.402	0.526	0.926	0.731	1.173
Female	2.3%	Ref.	Ref.	Ref.	Ref.	Ref.
<10 years	0	NA	NA	2.748E-09	2.748E-09	2.748E-09
11–25 years	2.7%	39.245	**<0.001**	10.695	5.095	22.448
26–44 years	3%	39.054	**<0.001**	9.696	4.755	19.771
45–59 years	1.7%	14.289	**<0.001**	4.244	2.006	8.979
>60 years	0.5%	Ref.	Ref.	Ref.	Ref.	Ref.
Sunny	2.2%	3.125	0.077	1.572	0.952	2.594
Cloudy	2.9%	5.528	**0.019**	1.959	1.118	3.432
Rainy	1.9%	Ref.	Ref.	Ref.	Ref.	Ref.
Morning	1.9%	3.235	0.072	0.795	0.620	1.021
Afternoon	2.6%	Ref.	Ref.	Ref.	Ref.	Ref.
Weekday	2.3%	0.864	0.353	1.139	0.866	1.498
Weekend	2.1%	Ref.	Ref.	Ref.	Ref.	Ref.
Earphones						
Male	2.1%	1.086	0.297	1.143	0.889	1.469
Female	1.9%	Ref.	Ref.	Ref.	Ref.	Ref.
<10 years	0.4%	0.031	0.860	0.868	0.180	4.193
11–25 years	4.9%	63.244	**<0.001**	22.610	10.484	48.761
26–44 years	2.1%	27.445	**<0.001**	7.745	3.601	16.659
45–59 years	0.6%	0.954	0.329	1.559	0.640	3.799
>60 years	0.4%	Ref.	Ref.	Ref.	Ref.	Ref.
Sunny	1.9%	0.028	0.867	0.964	0.628	1.480
Cloudy	2.1%	0.018	0.893	0.964	0.569	1.634
Rainy	2.8%	Ref.	Ref.	Ref.	Ref.	Ref.
Morning	2.3%	15.279	**<0.001**	1.691	1.299	2.200
Afternoon	1.7%	Ref.	Ref.	Ref.	Ref.	Ref.
Weekday	2.1%	3.927	**0.048**	1.367	1.003	1.862
Weekend	1.6%	Ref.	Ref.	Ref.	Ref.	Ref.
Holding						
Male	18%	81.970	**<0.001**	0.662	0.606	0.724
Female	27.2%	Ref.	Ref.	Ref.	Ref.	Ref.
<10 years	1.9%	5.803	**0.016**	0.439	0.225	0.858
11–25 years	34%	431.910	**<0.001**	15.049	11.654	19.434
26–44 years	28.5%	340.921	**<0.001**	10.141	7.930	12.968
45–59 years	17.7%	150.799	**<0.001**	5.004	3.870	6.470
>60 years	4.4%	Ref.	Ref.	Ref.	Ref.	Ref.
Sunny	23.3%	45.424	**<0.001**	1.910	1.583	2.306
Cloudy	24.8%	34.392	**<0.001**	1.907	1.537	2.367
Rainy	16.7%	Ref.	Ref.	Ref.	Ref.	Ref.
Morning	21%	6.72	**0.010**	0.887	0.810	0.971
Afternoon	24.6%	Ref.	Ref.	Ref.	Ref.	Ref.
Weekday	23.6%	8.657	**0.003**	1.163	1.052	1.287
Weekend	21.3%	Ref.	Ref.	Ref.	Ref.	Ref.
Slight smombie						
Male	1.5%	0.535	0.465	1.117	0.831	1.501
Female	1.3%	Ref.	Ref.	Ref.	Ref.	Ref.
<10 years	0.2%	0.828	0.363	0.381	0.047	3.050
11–25 years	2.2%	33.164	**<0.001**	9.083	4.287	19.247
26–44 years	1.6%	20.521	**<0.001**	5.361	2.592	11.085
45–59 years	1%	5.203	**0.023**	2.495	1.137	5.475
>60 years	0.5%	Ref.	Ref.	Ref.	Ref.	Ref.
Sunny	1.4%	0.810	0.368	1.302	0.733	2.315
Cloudy	1.4%	0.206	0.650	1.173	0.589	2.336
Rainy	1.5%	Ref.	Ref.	Ref.	Ref.	Ref.
Morning	1.2%	2.077	0.150	0.794	0.581	1.086
Afternoon	1.5%	Ref.	Ref.	Ref.	Ref.	Ref.
Weekday	1.5%	2.815	0.093	1.359	0.950	1.946
Weekend	1.2%	Ref.	Ref.	Ref.	Ref.	Ref.
Severe smombie						
Male	4.1%	0.036	0.850	1.017	0.852	1.215
Female	4.2%	Ref.	Ref.	Ref.	Ref.	Ref.
<10 years	0.6%	0.355	0.551	1.526	0.380	6.124
11–25 years	8.5%	85.852	**<0.001**	47.441	20.970	107.328
26–44 years	4.9%	55.255	**<0.001**	21.709	9.643	48.874
45–59 years	2%	19.551	**<0.001**	6.700	2.883	15.570
>60 years	0.4%	Ref.	Ref.	Ref.	Ref.	Ref.
Sunny	4.3%	14.538	**<0.001**	2.271	1.490	3.462
Cloudy	4.2%	8.341	**0.004**	2.019	1.253	3.252
Rainy	2.7%	Ref.	Ref.	Ref.	Ref.	Ref.
Morning	3.4%	9.768	**0.002**	0.740	0.613	0.894
Afternoon	4.7%	Ref.	Ref.	Ref.	Ref.	Ref.
Weekday	4.3%	3.151	0.076	1.206	0.981	1.483
Weekend	3.9%	Ref.	Ref.	Ref.	Ref.	Ref.

The meaning of the bold values is that they are statistically significant.

### Influencing Factors of Pedestrian Smartphone Use

#### Results From Bivariate Logistic Regression Analysis

The study used logistic regressions to analyze factors such as the gender and age of the pedestrians, weather, and time, which might influence the types of pedestrian smartphone use while crossing the road. Bivariate logistic regression analysis found that men were less likely to use smartphones while crossing the road than women (OR 0.757, 95% CI 0.699–0.819). In terms of age, teenagers were more likely to use smartphones than elderly pedestrians (OR 16.663, 95% CI 13.389–20.738), followed by young people (OR 10.248, 95% CI 8.309–12.639). In terms of weather, pedestrians were more likely to use smartphones on sunny days (OR 1.786, 95% CI 1.519–2.099) and cloudy days (OR 1.781 95% CI 1.477–2.147) than on rainy days. Pedestrians were also found to be more likely to use smartphones in the afternoon (OR 0.890, 95% CI 0.820–0.965) and on weekdays (OR 1.187, 95% CI 1.085–1.299). See Table 2.

#### Results From Multivariate Logistic Regression Analysis

For the multivariate logistic regression analysis, the results were more complicated, as shown in Table 3.

In the category of “talking”, teenagers were the most likely to be on a call while crossing the road (OR 10.695, 95% CI 5.095–22.448) and young people (OR 9.696, 95% CI 4.755–19.771) and middle-aged people (OR 4.244, 95% CI 2.006–8.979) followed.

Regarding the category of “earphones”, teenagers were the most likely to wear earphones while crossing the road (OR 22.610, 95% CI 10.484–48.761). Following these were young people (OR 7.745, 95% CI 3.601–16.659). Pedestrians were more likely to use earphones in the morning (OR 1.691, 95% CI 1.299–2.200) and on weekdays (OR 1.367, 95% CI 1.003–1.862).

With respect to “holding”, women were much more likely to hold smartphones than men (OR 0.662, 95% CI 0.606–0.724). Teenagers were more likely to cross the road with their smartphones in their hands than older people (OR 15.049, 95% CI 11.654–19.434). Young people were the second most likely to hold their phones (OR 10.141, 95% CI 7.930–12.968), followed by the middle-aged group (OR 5.004, 95% CI 3.870–6.470). Children were less likely to hold their smartphones than older people (OR 0.439, 95% CI 0.225–0.858). Pedestrians were more likely to hold smartphones in the afternoon (OR 0.887, 95% CI 0.810–0.971) and on weekdays (OR 1.163, 95% CI 1.052–1.287).

In the category of “slight smombie,” teenagers were more likely to be slightly more addicted to smartphone use than elderly pedestrians (OR 9.083, 95% CI 4.287–19.247). Young people aged 26 to 44 were the second most likely to be in the “slight smombie” category (OR 5.361, 95% CI 2.592–11.085), followed by the middle-aged group aged 45 to 59 (OR 2.495, 95% CI 1.137–5.475).

Similar to the situation in “slight smombie”, in the category of “severe smombie,” those aged 11 to 25 were more likely to use smartphones than elderly individuals (OR 47.441, 95% CI 20.970–107.328), and young people aged 26–44 years (OR 21.709, 95% CI 9.643–48.874) and middle-aged people aged 45–59 years (OR 6.700, 95% CI 2.883–15.570) followed. Pedestrians were more likely to be in the “severe smombie” category in the afternoon (OR 0.740, 95% CI 0.613–0.894).

### Smartphone Use With Intervention

There were 4,289 pedestrians who used smartphones in the first 11 days of the study without intervention, accounting for 32.8% of the observed people. In the next 11 days, with the visual and auditory intervention, 3,579 pedestrians were found to have used smartphones, accounting for 28.1% of the observed people. Through chi-square tests (*χ* ^*2*^ = 69.120, *p* < 0.001), smartphone usage rates with visual and auditory intervention were statistically lower than without intervention. See Supplementary Table S5.

The results on the effect of visual and auditory intervention on six categories showed that in comparison to those without intervention, the pedestrians with the intervention were more resistant to smartphones while crossing the road. The rates of “holding” (*χ* ^*2*^ = 49.228, *p* < 0.001), “slight smombie” (*χ* ^*2*^ = 9.047, *p* < 0.01), and “severe smombie” (*χ* ^*2*^ = 153.297, *p* < 0.001) decreased significantly. See Table 4.

**TABLE 4:** Multivariate logistic regression analysis with intervention as the independent variable. (Original research, smombie phenomenon, China, 2020).

	Usage Rates (%)	Wald χ^2^	*P*	OR	95% CI of OR	
					Lower	Upper
Talking						
With	2.2	1.115	0.291	0.914	0.774	1.080
Without	2.2	Ref.	Ref.	Ref.	Ref.	Ref.
Earphones						
With	3.2	28.209	**<0.001**	1.535	1.311	1.798
Without	2	Ref.	Ref.	Ref.	Ref.	Ref.
Holding						
With	19.9	49.140	**<0.001**	0.806	0.759	0.856
Without	23	Ref.	Ref.	Ref.	Ref.	Ref.
Slight smombie						
With	1.1	8.963	**0.003**	0.710	0.567	0.888
Without	1.4	Ref.	Ref.	Ref.	Ref.	Ref.
Severe smombie						
With	1.7	142.719	**<0.001**	0.375	0.319	0.440
Without	4.2	Ref.	Ref.	Ref.	Ref.	Ref.

The meaning of the bold values is that they are statistically significant.

**FIGURE 1:**
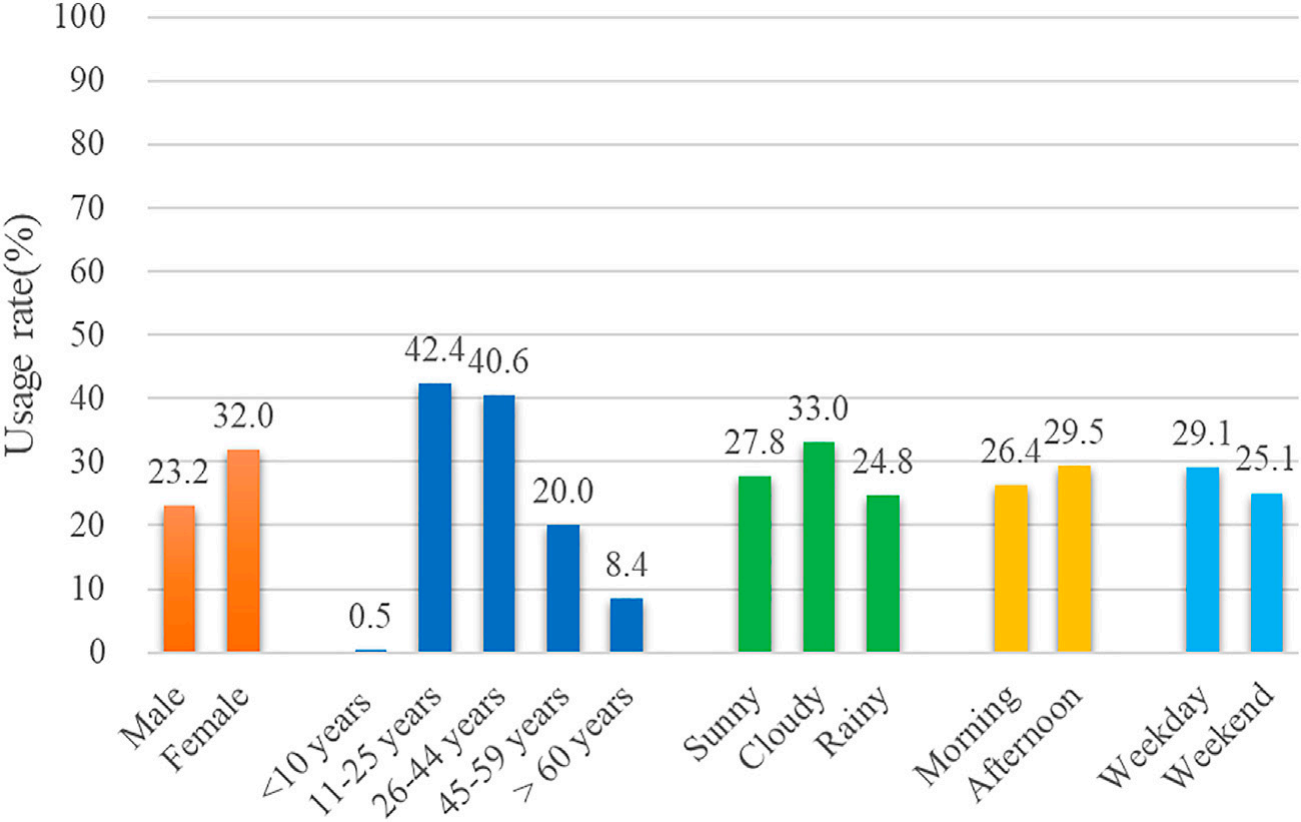
Smartphone usage rates with visual and auditory intervention (two categories). (Original research, smombie phenomenon, China, 2020). * “Unused” = “did not use smartphones”, “Used” = “used smartphones”.

The distribution of smartphone usage with intervention showed that usage rates were decreased both in women (*χ* ^*2*^ = 37.449, *p* < 0.001) and men (*χ* ^*2*^ = 33.414, *p* < 0.001) in comparison to those without intervention. The smartphone usage rates were decreased in all age groups (<10 years: *χ* ^*2*^ = 21.333, *p* < 0.001; 11–25 years: *χ* ^*2*^ = 33.758, *p* < 0.001; 45–59 years: *χ* ^*2*^ = 7.565, *p* < 0.001; >60 years: *χ* ^*2*^ = 5.629, *p* < 0.05), except for young people aged 26–45 (*χ* ^*2*^ = 0.253, *p* = 0.615). Smartphone usage rates were also found to decrease on sunny days (*χ* ^*2*^ = 66.992, *p* < 0.001). The usage rates with intervention in the morning (*χ* ^*2*^ = 17.524, *p* < 0.001) and afternoon (*χ* ^*2*^ = 49.818, *p* < 0.001) also decreased, as well as those on weekdays (*χ* ^*2*^ = 46.707, *p* < 0.001) and weekends (*χ* ^*2*^ = 46.707, *p* < 0.001). See Figure 1.

**FIGURE 2:**
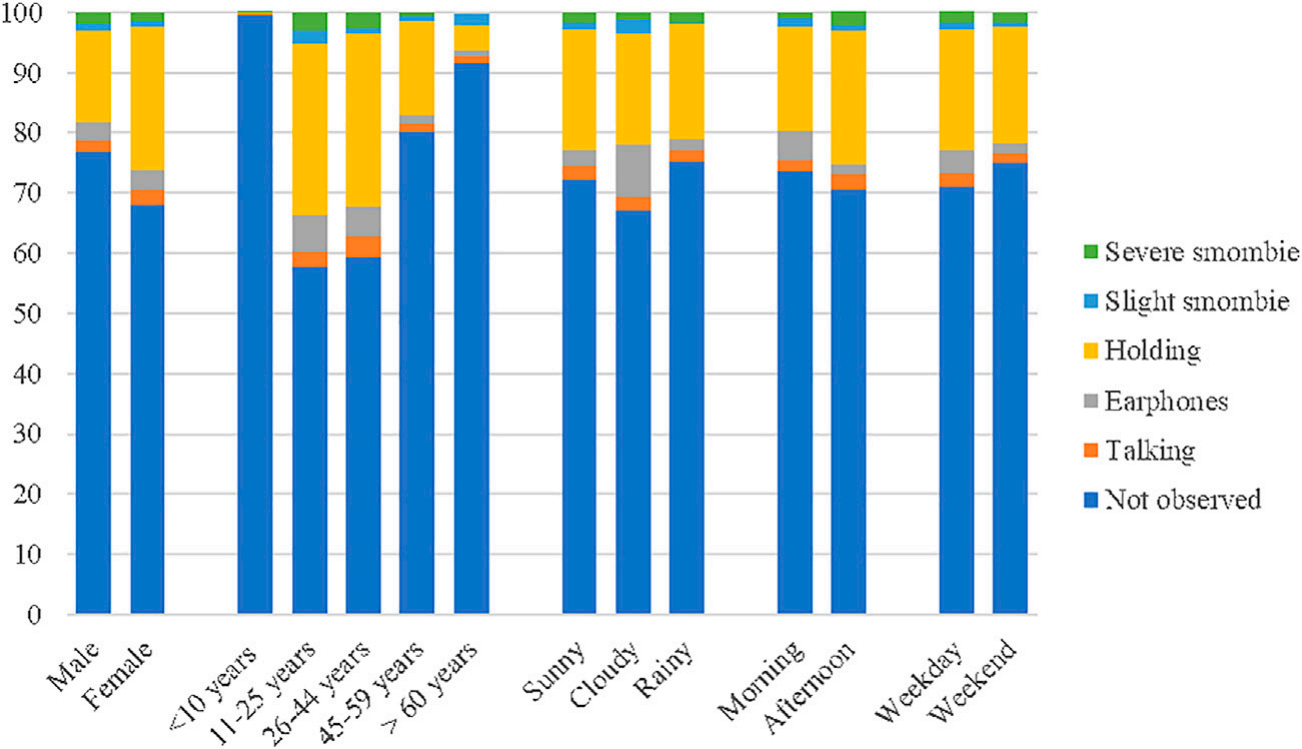
Usage rates of different types of smartphones use with visual and auditory intervention (six categories). (Original research, smombie phenomenon, China, 2020).

The “severe smombie” rates and the “holding” rates with the intervention were decreased in almost all conditions. The “slight smombie” rates were decreased both in women (*χ* ^*2*^ = 4.914, *p* < 0.05) and men (*χ* ^*2*^ = 4.144, *p* < 0.05), young people (*χ* ^*2*^ = 7.208, *p* < 0.01), on sunny (*χ* ^*2*^ = 10.901, *p* < 0.01) and rainy days (*χ* ^*2*^ = 6.045, *p* < 0.05), in the afternoon (*χ* ^*2*^ = 12.126, *p* < 0.001), and on weekdays (*χ* ^*2*^ = 4.751, *p* < 0.05) and weekends (*χ* ^*2*^ = 5.038, *p* < 0.05). The “talking” rates showed no significant change in all conditions. See Figure 2.

### The Effect of Combined Visual and Auditory Intervention

#### Results From Bivariate Logistic Regression Analysis

Using bivariate logistic regression analysis, we found that the visual and auditory intervention reduced the risk of pedestrian smartphone use while crossing the road (*χ* ^*2*^ = 69.013, *p* < 0.001). Pedestrians with visual and auditory intervention were less likely to use smartphones than those without intervention (OR 0.798, 95% CI 0.757–0.842). See Supplementary Table S5.

#### Results From Multivariate Logistic Regression Analysis

The results of the multivariate logistic analysis showed the impact of the intervention on different types of smartphone use. The visual and auditory intervention reduced the risk of “holding” (OR 0.806, 95% CI 0.759–0.856), “slight smombie” (OR 0.710, 95% CI 0.567–0.888), and “severe smombie” (OR 0.375, 95% CI 0.319–0.440). See Table 4.

## Discussion

The current study was a large-sample and multicenter observational study conducted in four cities in China with 25,680 pedestrians, which aimed to analyze the factors influencing pedestrian smartphone use while crossing roads, and the effect of visual and auditory intervention on smombie behavior. The results of this study showed that the proportion of pedestrian smartphone use while crossing roads was approximately one-third of the total number of observations, which is consistent with other studies [5, 6]. Factors influencing pedestrian smartphone use were gender, age, weather, and time. By observing pedestrian smartphone use behavior with and without intervention, the current study further demonstrated that visual and auditory intervention is an effective measure for reducing the smombie phenomenon. This study confirmed that smartphone use while crossing roads is common in China, and relevant interventions are urgently needed.

### Factors Influencing Pedestrian Smartphone Use

#### Effect of Different Influencing Factors on Pedestrian Smartphone Usage

In our study, the smartphone usage rates among the pedestrians crossing the road were 32.8%. Among the smartphone users, the usage rates of women were higher than those of men; teenagers had the highest prevalence of smartphone use while crossing the road, and pedestrians were more inclined to use smartphones while crossing the road, in the afternoon, on weekdays, and in good weather. In particular, women were more inclined to “hold” than men; teenagers were more inclined to use almost all types of smartphones use.

The bivariate logistic regression results showed that gender, age, weather, and time were the key factors influencing pedestrian smartphone use while crossing roads. In terms of gender, women were more likely to use their smartphones while crossing roads than men, which suggested that women might be more dependent on their smartphones. Previous studies have also confirmed the dependence of women on smartphones and attributed the phenomenon to their preference for social networks, in which reception and response to messages in a timely manner are necessary [23–25].

Of all the age groups, teenagers were most likely to use smartphones while crossing the road. Byington et al. [26] explored why teenagers use smartphones while crossing roads. “Understanding what friends are doing,” “replying to others in time,” and “finding important information” were proposed as the main reasons that might be associated with the psychological health of teenagers [27]. In addition to the teenagers, young people were also found to be the main group using their smartphones while crossing the road.

In terms of weather, the results were consistent with actual experience that pedestrians were more likely to use their smartphones on fine days. These results further confirmed why traffic accidents relative to pedestrians occurred more often on fine days [28, 29].

As we all have experienced, people are often short on time in the mornings when they go to school or work, but have plenty of time in the afternoon when they are leaving school or work. Therefore, it is not surprising that pedestrians are more likely to use smartphones in the afternoon. In addition, through our analysis of age, we found that the proportion of main users of smartphones (11–44 years) was much lower on weekends, which might explain the lower usage of smartphones on weekends compared to weekdays.

#### Different Types of Pedestrian Smartphone Use Were Affected by Different Factors

The “holding” rates were highest in all different types of smartphone use, followed by “severe smombie”. In both the teenage and young people groups, the behavior of “talking” was prominent.

Further multivariate logistic regression results found gender differences in the category of “holding”. Women were more likely to be “holding” their phones, which might be attributed to the season in which this study was conducted. We performed this study in summer, and the women’s clothes may have had no pockets for smartphones. Therefore, smartphones could only be held in their hand. Additionally, as women were more inclined to use smartphones for social networking [30], “holding” was more convenient for them to reply to messages quickly. The results also showed that teenagers were most inclined to use all types of smartphones, suggesting that teenagers had serious smartphone use behaviors while crossing the road, because interacting with others through smartphones was a priority for teenagers [31].

On rainy days, the behavior of “holding” and “severe smombie” was much less than that on non-rainy days, which might be due to the risk of potential harm caused by rain. Similar to the bivariate analysis, almost all smartphone use was more likely to occur in the afternoon than in the morning; and more likely to occur on weekdays than weekends. This was the first study on the influence of weather and time on pedestrian smartphone use while crossing roads.

### Effect of the Combined Visual and Auditory Intervention on Pedestrian Smartphone Use

With visual and auditory intervention, the smartphone usage rate decreased to 28.1%, which was significantly different from that without intervention. Specifically, the usage rates on “holding”, “slight smombie”, and “severe smombie” were significantly decreased compared to without intervention. Further analysis showed that our intervention was effective regardless of gender, age (except for young people), and time of day and week, suggesting that the combined visual and auditory intervention was generally available.

Through logistic regression analysis with and without intervention, we further confirmed the effectiveness of visual and auditory intervention in reducing smartphone use behavior while crossing roads. Because of distracted concerns, a single visual or auditory stimulus might be ignored by smombies [18, 19]. Therefore, we used a combined visual and auditory intervention, and the results demonstrated the preventive effect of visual and auditory intervention on “holding,” “slight smombie,” and “severe smombie.”

It is important to note that there was no significant difference in the category of “talking” with and without intervention. This might be related to the instantaneous nature of talking [5, 32]. Even when they were reminded of the danger of talking while crossing the road, the pedestrians continued ignoring their own safety to satisfy their needs for instant communication. This might be why our intervention was most unsuccessful in young people, who had the highest “talking” rate.

Unexpectedly, the usage of “earphones” by pedestrians increased rather than decreased with intervention. We thought that might be related to the intervention we performed. In pedestrians’ opinion, wearing earphones was relatively safe [20]. Thus, after being reminded not to use smartphones, the pedestrians put on earphones instead of holding phones in their hand.

### Strengths and Limitations

To the best of our knowledge, this study is the first large-sample, multicenter observation study on smartphone use while crossing roads. This study could reflect the current status of pedestrian smartphone use in China. Bivariate and multivariate logistic regression analyses were used to explore the influencing factors, such as gender, age, weather, and time. Moreover, visual and auditory intervention was used to prevent this behavior, and demonstrated an effect in reducing the use of smartphones while crossing the road.

There are also some limitations to our study. First due to the potential bias in estimating some variables (such as age), the results might have been influenced, even though we converted the continuous variables to categorical variables. Second, there was only one observer in each city, and the results might be biased. Moreover, this study did not conduct a statistical analysis of the regional effects. Then, there was no unified standard for the classification of smartphone types, and the devices that earphones were connecting to were unknown. In addition, the findings might be influenced by the seasons we conducted this study in. Moreover, the exclusion of pedestrians wearing watches in this study might not be easily distinguishable for observers, and therefore, the actual percentage of smartphone use might have been higher. In addition, the visual and auditory intervention was proven effective in the short term, but the long-term effects were not studied. Last, although visual and auditory intervention effectively reduced smartphone usage, more than a quarter of the pedestrians still used their smartphones while crossing the road. Thus, other interventions should be considered in the future, such as pedestrian bridges or dedicated “smartphone sidewalks”.

### Conclusion

In summary, through this observational study, we found that smartphone use while crossing roads is common in four cities in China. Gender, age, weather, and time were the main factors influencing pedestrian smartphone use while crossing roads. Moreover, our findings also verified the effect of the combined visual and auditory intervention on smartphone use. To our knowledge, this study is the first large-sample, multicenter observational study on pedestrian smartphone use and the intervention for it, which might provide a reference for future research.

## Data Availability

The data used to support the findings of this study are available from the corresponding author upon request.
