# Supplementary material for: Factors Influencing Pedestrian Smartphone Use and Effect of Combined Visual and Auditory Intervention on “Smombies”: A Chinese Observational Study
Source: Int J Public Health. 2022 Jun 22;67:1604601. doi: 10.3389/ijph.2022.1604601 (PMC9285399; doi:10.3389/ijph.2022.1604601)
Supplement: Supplementary file 1 [file DataSheet1.docx]

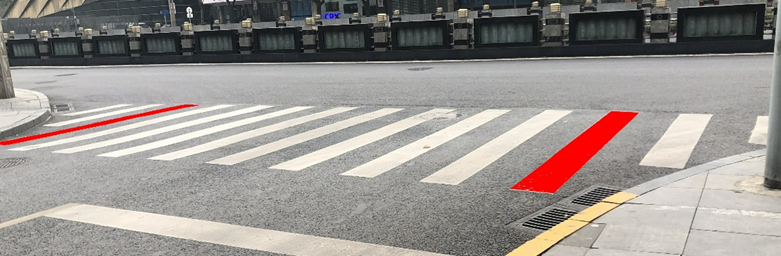


**Supplementary Figure 1** The fixed zebra sign. If the zebra crossing had different lengths, the longest one was chosen as the boundary line. (Original research, smombie phenomenon, China, 2020)


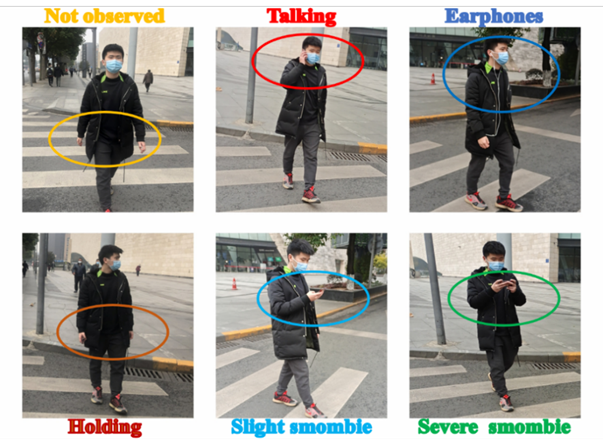


**Supplementary Figure 2** Types of pedestrian smartphone use were ranked from lower to higher use as follows: “Not observed”, “Talking”, “Earphones”, “Holding”, “Slight smombie”, and “Severe smombie.” (Original research, smombie phenomenon, China, 2020)


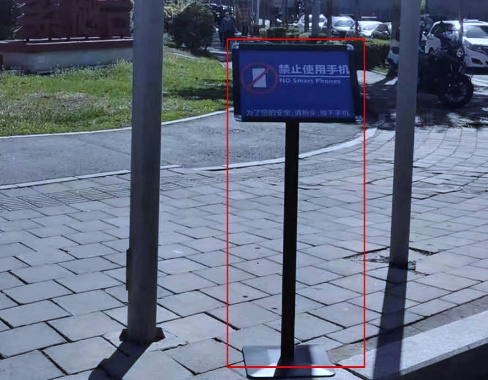


**Supplementary Figure 3** The safety warning sign (in the red box). In the last 11 days, the researchers put the sign on the sides of the road. (Original research, smombie phenomenon, China, 2020)

**Supplementary Table 1** Pairwise comparison results of different age groups (two categories). (Original research, smombie phenomenon, China, 2020)

|  | <10 years | | | 11-25 years | | | 26-44 years | | | 45-60 years | | >60 years | |
| --- | --- | --- | --- | --- | --- | --- | --- | --- | --- | --- | --- | --- | --- |
|  | χ^2^ | P | | χ^2^ | P | | χ^2^ | P | | χ^2^ | P | χ^2^ | P |
|  | 3.1% | | | 52.27% | | | 40.13% | | | 22.97% | | 6.14% | |
| <10 years |  | |  |  | |  |  | |  |  |  |  |  |
| 11-25 years | 415.476 | | <0.001 |  | |  |  | |  |  |  |  |  |
| 26-44 years | 278.233 | | <0.001 | 98.680 | | <0.001 |  | |  |  |  |  |  |
| 45-59 years | 108.238 | | <0.001 | 480.544 | | <0.001 | 251.749 | | <0.001 |  |  |  |  |
| >60 years | 7.070 | | 0.008 | 917.347 | | <0.001 | 666.804 | | <0.001 | 210.372 | <0.001 |  |  |

**Supplementary Table 2** Pairwise comparison results of different weather (two categories). (Original research, smombie phenomenon, China, 2020)

|  | Sunny | | Cloudy | | Rainy | |
| --- | --- | --- | --- | --- | --- | --- |
|  | χ^2^ | P | χ^2^ | P | χ^2^ | P |
|  | 33% | | 35% | | 26% | |
| Sunny |  |  |  |  |  |  |
| Cloudy | 3.520 | 0.061 |  |  |  |  |
| Rainy | 20.666 | <0.001 | 25.492 | <0.001 |  |  |

**Supplementary Table 3** Pairwise comparison results of different age groups (six categories). (Original research, smombie phenomenon, China, 2020)

| Variables  Types | <10 years | | | 11-25 years | | | 26-44 years | | | 45-60 years | | | >60 years | | |
| --- | --- | --- | --- | --- | --- | --- | --- | --- | --- | --- | --- | --- | --- | --- | --- |
|  | χ^2^ | | P | χ^2^ | P | | χ^2^ | P | | χ^2^ | P | | χ^2^ | P | |
| **Talking** | 0% | | | 2.7% | | | 3% | | | 1.7% | | | 0.5% | | |
| <10 years |  |  | |  | |  |  | |  |  | |  |  | |  |
| 11-25 years | 27.566 | <0.001 | |  | |  |  | |  |  | |  |  | |  |
| 26-44 years | 25.267 | <0.001 | | 0.413 | | 0.521 |  | |  |  | |  |  | |  |
| 45-59 years | 11.116 | 0.001 | | 24.099 | | <0.001 | 26.945 | | <0.001 |  | |  |  | |  |
| >60 years | 2.613 | 0.106 | | 60.348 | | <0.001 | 58.710 | | <0.001 | 17.039 | | <0.001 |  | |  |
| **Earphones** | 0.4% | | | 4.9% | | | 2.1% | | | 0.6% | | | 0.4% | | |
| <10 years |  |  | |  | |  |  | |  |  | |  |  | |  |
| 11-25 years | 27.566 | <0.001 | |  | |  |  | |  |  | |  |  | |  |
| 26-44 years | 13.431 | <0.001 | | 67.412 | | <0.001 |  | |  |  | |  |  | |  |
| 45-59 years | 0.612 | 0.434 | | 162.255 | | <0.001 | 43.172 | | <0.001 |  | |  |  | |  |
| >60 years | 0.028 | 0.866 | | 126.741 | | <0.001 | 37.776 | | <0.001 | 0.974 | | 0.324 |  | |  |
| **Holding** | 1.9% | | | 34% | | | 28.5% | | | 17.7 | | | 4.4% | | |
| <10 years |  |  | |  | |  |  | |  |  | |  |  | |  |
| 11-25 years | 284.417 | <0.001 | |  | |  |  | |  |  | |  |  | |  |
| 26-44 years | 204.332 | <0.001 | | 52.911 | | <0.001 |  | |  |  | |  |  | |  |
| 45-59 years | 89.287 | <0.001 | | 291.306 | | <0.001 | 164.176 | | <0.001 |  | |  |  | |  |
| >60 years | 6.426 | 0.011 | | 645.369 | | <0.001 | 493.674 | | <0.001 | 176.640 | | <0.001 |  | |  |
| **Slight smombie** | 0.2% | | | 2.2% | | | 1.6% | | | 1% | | | 0.5% | | |
| <10 years |  |  | |  | |  |  | |  |  | |  |  | |  |
| 11-25 years | 20.381 | <0.001 | |  | |  |  | |  |  | |  |  | |  |
| 26-44 years | 11.479 | 0.001 | | 9.049 | | 0.003 |  | |  |  | |  |  | |  |
| 45-59 years | 4.456 | 0.035 | | 32.915 | | <0.001 | 12.492 | | <0.001 |  | |  |  | |  |
| >60 years | 0.885 | 0.347 | | 46.570 | | <0.001 | 24.654 | | <0.001 | 5.463 | | 0.019 |  | |  |
| **Severe smombie** | 0.6% | | | 8.5% | | | 4.9% | | | 2% | | | 0.4% | | |
| <10 years |  |  | |  | |  |  | |  |  | |  |  | |  |
| 11-25 years | 78.132 | <0.001 | |  | |  |  | |  |  | |  |  | |  |
| 26-44 years | 34.101 | 0.001 | | 65.372 | | <0.001 |  | |  |  | |  |  | |  |
| 45-59 years | 7.197 | 0.007 | | 201.967 | | <0.001 | 67.238 | | <0.001 |  | |  |  | |  |
| >60 years | 0.364 | 0.546 | | 231.347 | | <0.001 | 107.699 | | <0.001 | 25.503 | | <0.001 |  | |  |

**Supplementary Table 4** Pairwise comparison results of different weather (six categories). (Original research, smombie phenomenon, China, 2020)

| Variables  Types | Sunny | | Cloudy | | Rainy | |
| --- | --- | --- | --- | --- | --- | --- |
|  | χ^2^ | P | χ^2^ | P | χ^2^ | P |
| **Talking** | 2.2% | | 2.9% | | 1.9% | |
| Sunny |  |  |  |  |  |  |
| Cloudy | 4.590 | 0.032 |  |  |  |  |
| Rainy | 0.833 | 0.361 | 4.132 | 0.042 |  |  |
| **Earphones** | 1.9% | | 2.1% | | 2.8% | |
| Sunny |  |  |  |  |  |  |
| Cloudy | 0.418 | 0.518 |  |  |  |  |
| Rainy | 1.818 | 0.178 | 0.409 | 0.523 |  |  |
| **Holding** | 23.3 % | | 24.8% | | 16.7% | |
| Sunny |  |  |  |  |  |  |
| Cloudy | 2.533 | 0.111 |  |  |  |  |
| Rainy | 21.976 | <0.001 | 25.174 | <0.001 |  |  |
| **Slight smombie** | 1.4% | | 1.4% | | 1.5% | |
| Sunny |  |  |  |  |  |  |
| Cloudy | 0.001 | 0.982 |  |  |  |  |
| Rainy | 0.041 | 0.840 | 0.033 | 0.855 |  |  |
| **Severe smombie** | 4.3% | | 4.2% | | 2.7% | |
| Sunny |  |  |  |  |  |  |
| Cloudy | 0.005 | 0.946 |  |  |  |  |
| Rainy | 7.301 | 0.007 | 5.887 | 0.015 |  |  |

**Supplementary Table 5** Bivariate logistic regression analysis with intervention as the independent variable. (Original research, smombie phenomenon, China, 2020)

|  | Usage rates | *Wald* χ^2^ | *P* | OR | 95% CI of OR | |
| --- | --- | --- | --- | --- | --- | --- |
|  |  |  |  |  | Lower | Upper |
| With | 28% | 69.013 | **<.001** | 0.798 | 0.757 | 0.842 |
| Without | 32.8% | *Ref.* | *Ref.* | *Ref.* | *Ref.* | *Ref.* |
